# Local Path Planning for Mobile Robots Based on Fuzzy Dynamic Window Algorithm

**DOI:** 10.3390/s23198260

**Published:** 2023-10-05

**Authors:** Ying Sun, Wenlu Wang, Manman Xu, Li Huang, Kangjing Shi, Chunlong Zou, Baojia Chen

**Affiliations:** 1 Key Laboratory of Metallurgical Equipment and Control Technology of Ministry of Education, Wuhan University of Science and Technology, Wuhan 430081, China; sunying65@wust.edu.cn (Y.S.); shikangji9@wust.edu.cn (K.S.); 2 Hubei Key Laboratory of Mechanical Transmission and Manufacturing Engineering, Wuhan University of Science and Technology, Wuhan 430081, China; 3 Research Center for Biomimetic Robot and Intelligent Measurement and Control, Wuhan University of Science and Technology, Wuhan 430081, China; 4 College of Computer Science and Technology, Wuhan University of Science and Technology, Wuhan 430081, China; huangli82@wust.edu.cn; 5 Hubei Province Key Laboratory of Intelligent Information Processing and Real-Time Industrial System, Wuhan University of Science and Technology, Wuhan 430081, China; 6 College of Mechanical Engineering, Hubei University of Automotive Technology, Shiyan 442002, China; zouchunlongh_hapm@163.com; 7 Hubei Key Laboratory of Hydroelectric Machinery Design & Maintenance, China Three Gorges University, Yichang 443005, China

**Keywords:** dynamic windowing algorithm, efficient human–robot collaboration, mobile robot, dynamic path planning, fuzzy control

## Abstract

Due to the increased employment of robots in modern society, path planning methods based on human–robot collaborative mobile robots have been the subject of research in both academia and industry. The dynamic window approach used in the research of the robot local path planning problem involves a mixture of fixed weight coefficients, which makes it hard to deal with the changing dynamic environment and the issue of the sub-optimal global planning paths that arise after local obstacle avoidance. By dynamically modifying the combination of weight coefficients, we propose, in this research, the use of fuzzy control logic to optimize the evaluation function’s sub-functions and enhance the algorithm’s performance through the safe and dynamic avoidance of obstacles. The global path is introduced to enhance the dynamic window technique’s ability to plan globally, and important points on the global path are selected as key sub-target sites for the local motion planning phase of the dynamic window technique. The motion position changes after local obstacle avoidance to keep the mobile robot on the intended global path. According to the simulation results, the enhanced dynamic window algorithm cuts planning time and path length by 16% and 5%, respectively, while maintaining good obstacle avoidance and considering a better global path in the face of various dynamic environments. It is difficult to achieve a local optimum using this algorithm.

## 1. Introduction

With the development of robot technology, HRC-based robots are widely used in autonomous navigation, inspection and exploration, agricultural machinery services, logistics and warehousing, and other fields [1,2,3,4]. Map creation and localization, path planning, and controlled movement are the three main components of autonomous navigation for mobile robots [5,6,7].

One of the most important of these is the path planning of mobile robots, which can be divided into global path planning and local path planning depending on the availability of environmental information. It is necessary to be familiar with the map and the obstacles of an area to plan a global path. Global paths can only be applied to static environments, but this is not in line with the actual motion environment of mobile robots, and current industrial applications also require mobile robots to be able to achieve local dynamic obstacle avoidance while considering a better global path [8,9].

Therefore, local path planning and dynamic obstacle avoidance algorithms for mobile robots must be studied [10,11,12]. To execute real-time obstacle avoidance and online planning based on the local environment information, local path planning uses many sensors, which gather local environment information in real time while the robot is moving.

Most human labor has been replaced by robots, yet accessible human–robot collaboration still requires technological advances. In this paper, the dynamic window algorithm is improved to solve robots’ path planning problems more effectively. Fuzzy control is used to dynamically adjust the combination of weight coefficients, and the evaluation function of the dynamic window approach is further improved to enhance its dynamic obstacle avoidance capability. Finally, the global path is introduced, and the position of the mobile robot is adjusted after local obstacle avoidance to achieve better dynamic obstacle avoidance and human–robot collaboration.

The rest of the paper is organized as follows: Section 2 presents the relevant research on path planning methods in the past few years. Section 3 analyzes the dynamic window algorithm to obtain directions for improvement, proposes a dynamic window algorithm based on fuzzy control [13], and introduces the globally optimal path [14]. Section 4 compares the proposed algorithm with previous algorithms to demonstrate the effectiveness and advancement of the proposed algorithm. Section 5 concludes the paper.

## 2. Related Work

The local path planning methods that are the most effective and commonly used include the D* algorithm, artificial potential field method [15], fuzzy logic method [16], dynamic window method [17], etc. The Dynamic A* (D*) algorithm is a dynamic planning algorithm proposed by Stentz et al., based on the A* algorithm, which can quickly replan part of the local path when the environment changes without replanning the whole path, thus reducing the computational effort and meeting real-time requirements. Khatib was the first to propose the artificial potential field (APF) method [18] and to apply it to path planning. The artificial potential field method is easy to calculate, more effective in real time, safer, and does not collide with obstacles [19]. The fuzzy logic method (fuzzy logic) is based on expert experience and establishes an accurate correspondence mapping of the external environment for robot motion control, thus enabling local path planning [20,21]. Fuzzy logic has certain advantages in dealing with complex environments and planning paths in real time.

The dynamic window approach (DWA) takes into account the kinematics and dynamics of the mobile robot during local path planning. It converts the problem of local path planning into an optimization problem with velocity constraints. Compared with other local path planning algorithms, the dynamic window approach initially incorporates the mechanical characteristics of the mobile robot itself. It takes into account the environmental restrictions related to velocity and other factors, which grants it distinct advantages in local path planning. Consequently, the generated trajectories are more in line with the requirements of the actual control of the robot [22]. It has been extensively studied and applied in indoor complex dynamic environment motion planning [23]. Chang et al. [24] improved the evaluation function of the DWA algorithm by integrating the Q-learning algorithm. The improved DWA algorithm primarily enhances and extends the evaluation function, while introducing two additional evaluation functions to enhance the algorithm’s performance. Kiss et al. [25] introduced a global dynamic window navigation scheme, leveraging an unweighted objective function rooted in model predictive control for the problem that the DWA algorithm cannot pass narrow bands when formulating optimal paths. Henkel et al. [26] took the mobile robot motion process as an entry point to improve the dynamic planning efficiency of the algorithm and experimentally verified that the improved local path planner can further reduce energy consumption.

Compared with the above local path planning algorithms, the D* algorithm exhibits notable challenges, such as low search efficiency, high cost, and making multiple turns in a tiny region. The artificial potential field method tends to fall into local optimality and can suffer from problems such as unreachable targets and path fluctuations, when employed across diverse environmental settings. This requires a more reasonable potential field function according to the given scene, and the wide application is not strong [27,28]. The fuzzy logic method relies on expert experience and lacks flexibility [29]. The dynamic window method exhibits inefficacy when encountering atypical obstacles, such as “L”- or “C”-shaped obstacles.

However, the dynamic window algorithm takes into account the robot’s motion performance in local path planning [30,31]. This is more in line with the actual motion of the mobile robot. It can greatly improve the performance of the algorithm if combined with other algorithms [32,33].

## 3. Dynamic Windowing Algorithm Based on Fuzzy Control

### 3.1. Dynamic Window Algorithm Analysis and Improvement Direction

#### 3.1.1. Dynamic Window Algorithm Analysis

The basic principle of the dynamic window approach (DWA) is based on the magnitude and direction of the recorded velocity at a certain time. The trajectory of the mobile robot during the subsequent time interval is simulated simultaneously [34]. The generated trajectories are assessed and an evaluation index is developed. The velocity associated with the trajectory with the largest value of the evaluation function is calculated to ascertain the current motion state of the mobile robot. The core idea of the dynamic window method comprises three main components: motion model establishment, velocity space sampling, and trajectory evaluation function design [35].

(1) Modeling the kinematics of mobile robots

**Figure 1:**
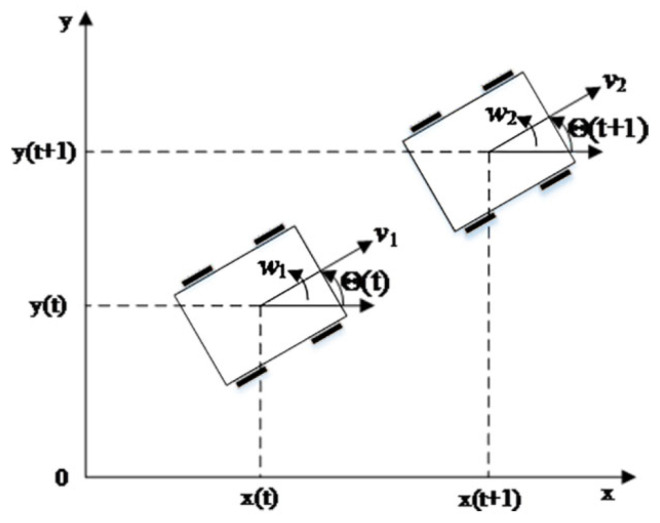
A kinematic model for mobile robots.

In the dynamic window method, the optimal combination of linear and angular positions in each simulation cycle is calculated based on the motion model of the mobile robot. Therefore, the kinematic model of the mobile robot needs to be established first [36,37]. The fundamental kinematic models of mobile robots are both non-holonomic and omnidirectional motion. The mobile robot platform utilized in this section is a robot with non-omnidirectional motion, featuring only two types of motion, forward and steering. The kinematic model of the mobile robot is shown in Figure 1, where four drive wheels realize the forward movement of the mobile robot, and the steering of the mobile robot is realized by the differential rotation of the two front drive wheels.

The mobile robot’s trajectory can be approximated as uniform linear motion during the two sampling intervals before and after, given that the sampling period Δ*t* is typically small. Accordingly, the change in the mobile machine’s position and heading angle in the subsequent time intervals can be expressed as:

(1) $$\left\{\begin{array}{c} \Delta x=v_{t}\cdot \Delta t\cdot \cos\left(\theta_{t}\right)\\ \Delta y=v_{t}\cdot \Delta t\cdot \sin\left(\theta_{t}\right)\\ \Delta \theta =w\cdot \Delta t \end{array}\right.$$

(2) $$\left\{\begin{array}{c} x\left(t+1\right)=x\left(t\right)+v_{t}\cdot \Delta t\cdot \cos\left(\theta_{t}\right)\\ y\left(t+1\right)=y\left(t\right)+v_{t}\cdot \Delta t\cdot \sin\left(\theta_{t}\right)\\ \theta \left(t+1\right)=\theta \left(t\right)+w\cdot \Delta t \end{array}\right.$$

(2) Sampling speed space

The dynamic window algorithm necessitates determining the range of linear velocity [*v*_min_, *v*_max_] and angular velocity [*w*_min_, *w*_max_] within the current simulation period before simulating the motion trajectory of each set of sampled velocity combinations (*v*, *w*). The sampled velocities must take into account the maximum and minimum velocities achievable by the mobile robot in a sampling period, the limitations of mechanical properties on the velocities, and the limitations of obstacle avoidance on the velocity limitations. In summary, the velocity sampling space of the dynamic window algorithm comprises three components. The first component encompasses the maximum and minimum velocity that the mobile robot can reach. The second component encompasses the maximum and minimum velocity that the mobile robot can reach within a simulation period limited by its mechanical performance. The third component involves selecting velocities to ensure that the mobile robot can stop its motion before colliding with an obstacle.

(1) Kinematic constraints

The maximum and minimum speed limit of the mobile robot itself is:

(3) $$V_{s}=\left\{\left.\left(v,w\right)\right|v\in \left[v_{\min},v_{\max}\right]\Lambda w\in \left[w_{\min},w_{\max}\right]\right\}$$

In the formula, *v_min_*, *v_max_*—minimum and maximum linear speed of the robot; *w_min_*, *w_max_*—minimum and maximum angular velocity of the robot.

(2) Mechanical performance constraints

The output torque of the mobile robot’s motor is constrained, resulting in limitations on both maximum acceleration and maximum deceleration for both linear and angular velocities [38]. The set of velocities *V_d_* that is constrained by the maximum acceleration and deceleration in one simulation period should satisfy the following:

(4) $$V_{d}=\left\{\left.\left(v,w\right)\right|v\in \left[v_{c}-{\overset{•}{v}}_{b}\cdot \Delta t,v_{c}+{\overset{•}{v}}_{a}\cdot \Delta t\right]\Lambda w\in \left[w_{c}-{\overset{•}{w}}_{b}\cdot \Delta t,w_{c}+{\overset{•}{w}}_{a}\cdot \Delta t\right]\right\}$$

In the formula,

*v*_c_, *w*_c_—linear and angular velocities of the mobile robot in its current motion;

${\overset{•}{v}}_{a}$, ${\overset{•}{v}}_{b}$—maximum acceleration and maximum deceleration of the linear velocity of the mobile robot;

${\overset{•}{w}}_{a}$, ${\overset{•}{w}}_{b}$—maximum acceleration and maximum deceleration of the angular velocity of the moving robot.

(3) Security constraints

To uphold the safety measures in the dynamic window method of obstacle avoidance, the sampled velocity should allow the mobile robot to come to a complete stop before reaching the obstacle. Thus, the sampled velocity space must also be constrained by the braking distance [39]. Therefore, the set of velocities *Va* sampled in a period, constrained by the maximum deceleration that enables the mobile robot to come to a stop prior to colliding with an obstacle, should satisfy the following:

(5) $$V_{a}=\left\{\left.\left(v,w\right)\right|v\leq \sqrt{2\cdot dist\left(v,w\right)\cdot {\overset{•}{v}}_{b}}\Lambda w\leq \sqrt{2\cdot dist\left(v,w\right)\cdot {\overset{•}{w}}_{b}}\right\}$$

In the formula, *dist*(*v*,*w*)—the shortest distance to the obstacle on the trajectory corresponding to speed (*v*,*w*).

Combining the above three speed constraints, the ultimate selection for the admissible velocity is the intersection of these sets. Let *Vr* denote the set of admissible velocities; thus, *Vr* should satisfy the following:

(6) $$V_{r}=V_{s}\cap V_{d}\cap V_{a}$$

(3) Design trajectory evaluation function

The purpose of the evaluation function is to choose the optimal trajectory. Varying speeds will give different predicted trajectories, consequently yielding evaluation function values of varying magnitudes. The primary criterion in formulating the evaluation function is to ensure that the mobile robot avoids obstacles as much as possible in local path planning while advancing in the direction of the target point at the fastest speed.

The equation of the designed evaluation function is:

(7) $$G\left(v,w\right)=\sigma \left(\alpha \cdot Heading\left(v,w\right)+\beta \cdot Dist-obstacle\left(v,w\right)+\gamma \cdot Velocity\left(v,w\right)\right)$$

In the formula:

*Heading*(*v*, *w*)—a target azimuth evaluation function indicating the azimuthal deviation between the end direction of the simulated trajectory and the target point at the current velocity;

*Obstacle-Distance*(*v*, *w*)—evaluation function of the distance between the obstacle and the cart;

*Velocity*(*v*, *w*)—evaluation function of the velocity magnitude of the current motion;

*α, β, γ*—the weight coefficients of the three sub-functions; *α, β, γ*;

*σ*—the coefficients normalized to the three components of the trajectory evaluation function are generally constants.

Among them:

(8) $$heading\left(v,w\right)=180^{\circ }-\theta$$

(9) $$dist\left(v,w\right)=\left\{\begin{array}{c} d,d<d_{\max}\\ d_{\max},d\geq d_{\max} \end{array}\right.$$

After obtaining the results of the evaluation function of the above three components, each component should be normalized. Subsequently, they are combined, aiming to make the trajectory smoother and avoiding a certain part accounting for too large a proportion. The normalization process entails dividing each term by the sum of the terms, ensuring that the three components of the trajectory evaluation function are normalized within the interval [0, 1]. The normalization calculation formula is as follows:

(10) $$\left\{\begin{array}{c} normal\_heading\left(i\right)=\frac{heading\left(i\right)}{\sum_{i=1}^{n}heading\left(i\right)}\\ normal\_dist\left(i\right)=\frac{dist\left(i\right)}{\sum_{i=1}^{n}dist\left(i\right)}\\ normal\_velocity\left(i\right)=\frac{velocity\left(i\right)}{\sum_{i=1}^{n}velocity\left(i\right)} \end{array}\right.$$

In Equation (10), *n*—all sample traces in one simulation cycle; *i*—current simulation trajectory to be evaluated.

The dynamic window method has the capability to engage in real-time obstacle avoidance and online path planning by leveraging locally detected environmental information. The dynamic obstacle avoidance performance is superior, resulting in a smoother planned motion trajectory that aligns closely with the actual motion trajectory of the mobile robot. However, the algorithm has a short sampling. It can only compute based on the current window, lacking global information from the starting point to the target point. Therefore, it is prone to falling into the local optima, and the resulting path is often not the optimal global path [40].

#### 3.1.2. Dynamic Window Algorithm Improvement Direction

The magnitude of appropriate weight coefficients, denoted as *α*, *β*, and *γ*, in the trajectory evaluation function *G* (*v, w*) of the dynamic window method is pivotal in the selection of an optimal trajectory. While weighting coefficients in the dynamic window method are typically determined through experimental exploration for optimal constant combination, the real operational environments for mobile robots are inherently uncertain. Consequently, achieving an ideal combination of constant *α*, *β*, and *γ* that caters to practical requirements can pose a challenge. Based on an extensive set of simulation comparison experiments, Fox D. et al. [41] concluded that the algorithm exhibits enhanced path planning and dynamic obstacle avoidance capabilities when *α* is taken as 0.8, *β* as 0.1, and *γ* as 0.1. The experimental results demonstrated the mobile robot’s adeptness in navigating through a challenging environment and avoiding obstacles. However, the benefit of dynamic obstacle avoidance through the dynamic window method is notably diminished in complicated scenarios, often leading to a tendency to converge towards a local optimum.

**Figure 2:**
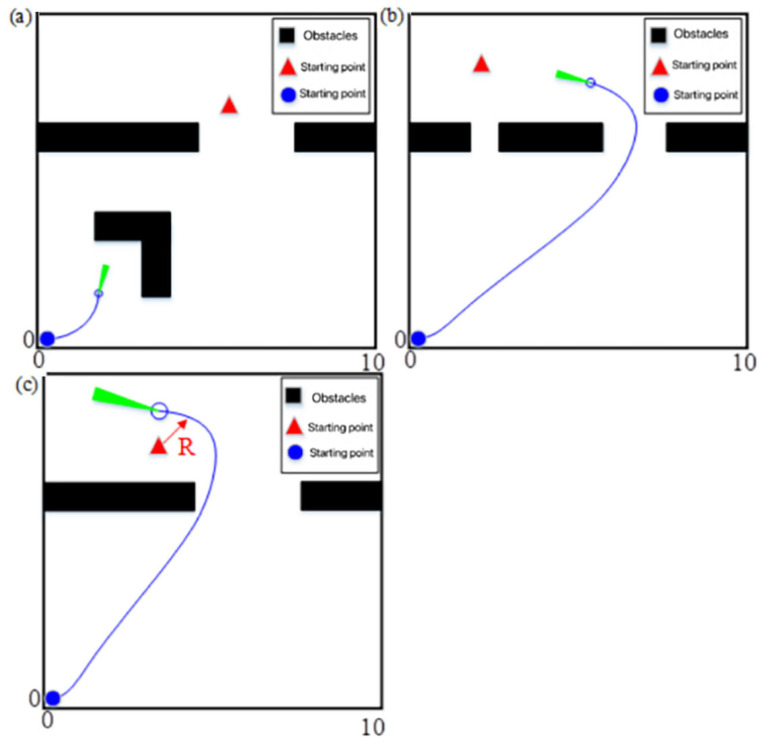
The main problems faced by the dynamic window method when using different combinations of fixed weights: (**a**) *α* = 0.8, *β* = 0.1, *γ* = 0.1; (**b**) *α* = 0.1, *β* = 0.8, *γ* = 0.1; (**c**) *α* = 0.1, *β* = 0.1, *γ* = 0.8.

Analysis of the problems of the dynamic window method:

(1) When encountering “L”- or “C”-type obstacles positioned along the path between the mobile robot and the target point, it is easy to fall into the local optimum. When *α* = 0.8, *β* = 0.1, *γ* = 0.1, the target azimuth weight *α* is more extensive, and the mobile robot will head toward the target point. However, the mobile robot will be blocked by the trap-type obstacle on the line between the mobile robot and the target point. It may not be able to bypass the obstacle because the obstacle avoidance weight is too small, thus falling into the local optimum, as shown in Figure 2a.
(2) Prioritizing wider channels leads to an increase in path length. When *α* = 0.1, *β* = 0.8, *γ* = 0.1, the mobile robot is more capable of avoiding obstacles. However, it may cause the mobile robot to ignore narrower passable passages and choose more expansive passages instead. While this approach maximizes safety, it will lead to a significant increase in the length of the path found by the algorithm, making it difficult to achieve an optimized trajectory, as shown in Figure 2b.
(3) The speed weight is too high and the target point represents a reachability problem. When *α* = 0.1, *β* = 0.1, *γ* = 0.8, the mobile robot will have a higher bias towards speed and choose the running trajectory with a more significant speed, which can save time to a certain extent. However, it may lead to the mobile robot failing to reach the target point because the speed is too high, the turning radius is larger, and the target point is missed, as shown in Figure 2c.

Due to the aforementioned challenges, it can be said that the dynamic window method’s improvement path is mostly focused on the trajectory function, which largely entails improving the sub-function and dynamically adjusting the combination of the weight coefficients. The dynamic window method’s capacity for local obstacle avoidance can be enhanced by further sub-function enhancement. The dynamic adjustment can solve challenges related to lengthy path trajectories and elusive goals. They play a crucial role in preventing the dynamic window technique from falling into local optimum and enable the application of the dynamic window algorithm to more complicated and variable dynamic environments.

### 3.2. Dynamic Windowing Algorithm Based on Fuzzy Control

#### 3.2.1. Fuzzy Control Dynamically Adjusts the Combination of Weight Coefficients

The dynamic window method and its improved algorithms often rely on fixed evaluation function weight parameters when dealing with varying obstacle distributions, leading to unsatisfactory path planning outcomes of the algorithm in some scenarios [42].

Allowing for an infinite combination of evaluation function weights will inevitably cause the dynamic window algorithm to struggle in planning the optimal path under certain specific circumstances. This can lead to the problems of falling into the local optimum and unreachable objectives. To address the above problems, this paper introduces a fuzzy control method based on the dynamic window algorithm. This method uses the fuzzy control method to dynamically adjust the combination of weight coefficients in the evaluation function. The aim is to better adapt to the complex and changing environment and improve the dynamic obstacle avoidance capability of the algorithm.

The construction of a fuzzy controller, foundational to fuzzy control, primarily entails four processes: input fuzzification, fuzzy control rule establishment, fuzzy inference, and inverse fuzzification. The most crucial of the four steps is fuzzy control rule establishment.

The fuzzy controller designed in this section incorporates two inputs and three outputs. The inputs of the fuzzy controller are the distance from the mobile robot to the target point, Dist-Goal, and the distance from the mobile robot to the nearest obstacle, Dist-Obstacle. It can improve the safety of the planned path and significantly reduce the likelihood of collision with obstacles, which is a critical parameter in dynamic obstacle avoidance. The outputs consist of three weights: *α* for the target azimuth evaluation function, *β* for the distance evaluation function between the mobile robot and the obstacle, and *γ* for the velocity evaluation function of the mobile robot.

**Figure 3:**
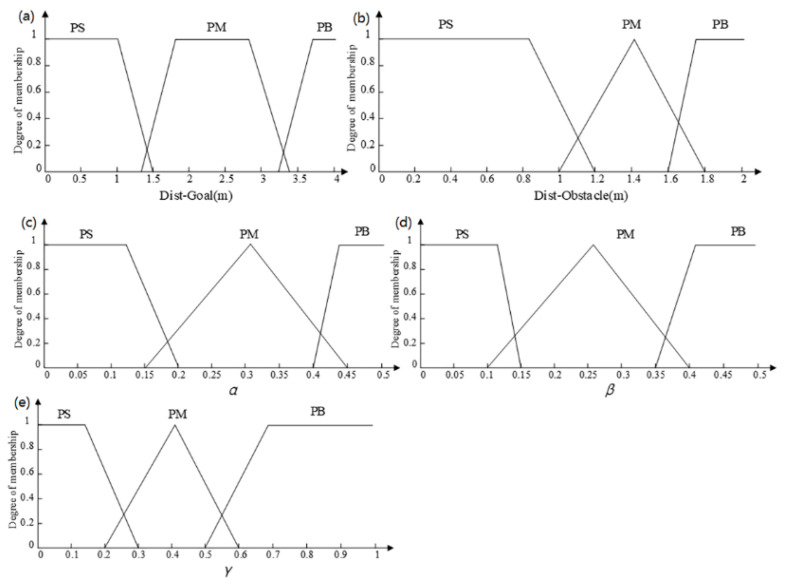
Affiliation functions of the input variables and output variables: (**a**) Input variable Dist-Goal; (**b**) input variable Dist-Obstacle; (**c**) output variable *α*; (**d**) output variable *β*; (**e**) output variable *γ*.

The input and output variables of the fuzzy controller use continuous-type theoretical domains, and trapezoidal and triangular functions are used as the affiliation functions. The domain of the input variable Dist-Goal is set to [0, 4], and the fuzzy sets are {PS, PM, PB}, which correspond to the near, medium, and far distances, respectively, max{Dist-Goal} = 4. The input variable Dist-Obstacle is set to [0, 2] with fuzzy sets {PS, PM, PB} corresponding to near, medium, and far distances, respectively, max{Dist-Obstacle} = 2. The graph of the affiliation function of the input variables is shown in Figure 3.

The theoretical domains for the output variables *α*, *β*, and *γ* are uniformly defined as [0, 1]. Correspondingly, the corresponding fuzzy sets are all {PS, PM, PB}, which represent {positive small, positive medium, positive large}, respectively. The affiliation function graph for the input variables is shown in Figure 3. The theoretical domains for the output variables *α*, *β*, and *γ* are uniformly defined as [0, 1]. Correspondingly, the corresponding fuzzy sets are all {PS, PM, PB}, which represent {positive small, positive medium, positive large}, respectively.

The core of a fuzzy controller is a collection of fuzzy rules, based on the affiliation function. These rules map exact values in an argument’s domain to various fuzzy sub-sets so that fuzzy control can be achieved by substituting fuzzy variable values such as “positive large”, “positive medium”, and “positive small” for exact values [43]. Table 1 presents the fuzzy rule table created by the dynamic window method’s algorithmic features based on the actual motion of the mobile robot, and the following fuzzy rule design principles are used.

(1) When the distance between the mobile robot and the target point and the proximity to the nearest obstacle are considerable, higher values of *α* and *γ* and smaller values of *β* should be selected at this time. This configuration encourages the mobile robot to prioritize trajectory with higher speeds, aiming toward the target point to shorten the distance to the target point quickly.
(2) In scenarios where the distance between the mobile robot and the target point is significant, and the proximity to the nearest obstacle is minimal, smaller values of *α* and *γ* and larger values of *β* should be chosen at this time. This approach encourages the mobile robot to favor a trajectory with low speed and far away from the obstacle to avoid colliding with the obstacle.
(3) In cases where the distance between the mobile robot and the target point is small, and the distance to the nearest obstacle is significant, smaller values of *β* and *γ* and larger values of *α* should be chosen at this time. This strategy encourages the mobile robot to prioritize the trajectory with low speed, aiming toward the target point to avoid missing the target point.
(4) In instances where the distance between the mobile robot and the target point is small and the proximity to the nearest obstacle is close, a more significant value of *α*, a moderate value of *β*, and a smaller value of *γ* should be chosen. This configuration steers the mobile robot to favor a trajectory with low speed and toward the target point, while avoiding collision with the obstacle.

After obtaining the output variables, the fuzzy vector is generated using the Mamdain-type fuzzy inference method. Subsequently, precise output control values are derived through defuzzification using the center of gravity method. This allows the coefficients of the weights of each component of the trajectory evaluation function to be calculated.

#### 3.2.2. Dynamic Window Algorithm Evaluation Sub-Function Optimization

In the dynamic environment, the mobile robot typically approaches obstacles closely to detect dynamic obstacles and activate the judgment function. However, the deceleration of the mobile robot is limited. If the robot or the obstacle moves too swiftly, a collision becomes probable, which is unacceptable. Therefore, there is a need to optimize the three evaluation sub-functions of the traditional dynamic window method to a certain extent to enhance its safe and effective dynamic obstacle avoidance capability.

(1) Considering the kinematic constraints, motor dynamics constraints, and safety constraints of the mobile robot, the velocity range within a specific time Δ*t* is limited. Assuming Δ*t* is sufficiently small, both linear velocity and angular velocity are maintained as constant within this duration. The linear and angular velocities in the velocity space are sampled, and the trajectory is determined based on the magnitude and direction of the linear and angular velocities in time Δ*t*. The angle of the good area is restricted in alignment with the determined trajectory. The area with more obstacles is avoided as much as possible, and the azimuth evaluation function *Heading*(*v*, *w*) is limited according to the angle of the good area.
(2) The distance evaluation function, which assesses the distance between the mobile robot and the obstacles, undergoes enhancement by incorporating a function that accounts for the distance between the mobile robot and the sub-target point to obtain the new distance evaluation function equation:

(11) $$Dist\left(v,w\right)=a\cdot Dist-obstacle\left(v,w\right)+b\cdot Dist-goal\left(v,w\right)$$

In the formula, a, b—constants.

(3) The velocity evaluation function, *Velocity*(*v*, *w*), is optimized to ensure that the velocity automatically diminishes when it is close to a dynamic obstacle. However, the reduction is controlled to avoid reaching zero velocity, achieving a safer and faster effect of avoiding dynamic obstacles. In achieving this objective, the introduction of the velocity difference between the mobile robot and the dynamic obstacle is to be considered. Consequently, the new velocity evaluation function formula is obtained as:

(12) $$New-velocity\left(v,w\right)=m\cdot Velocity\left(v,w\right)+n\cdot \Delta Velocity\left(v,w\right)$$

In the formula, Δ*Velocity*(*v*, *w*)—difference in speed between the mobile robot and dynamic obstacle; m, n—constants.

The final optimized evaluation function equation is:

(13) $$G\left(v,w\right)=\sigma \left[\alpha \cdot Heading\left(v,w\right)+\beta \cdot Dist\left(v,w\right)+\gamma \cdot New-velocity\left(v,w\right)\right]$$

**Figure 4:**
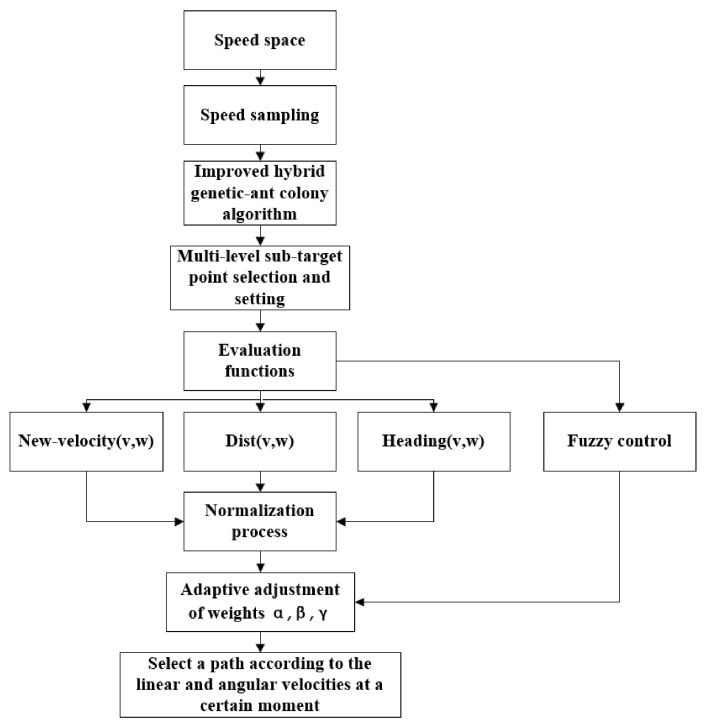
Flowchart for improving the dynamic window method.

The final flowchart of the improved dynamic window method is shown in Figure 4. The algorithm flow steps are:

(1) The velocity range within a specific time Δ*t* is constrained in accordance with the mobile robot’s kinematic constraints, motor dynamics constraints, and safety constraints. Under the assumption that the time Δ*t* is sufficiently short, both linear velocity and angular velocity remain constant within the time Δ*t*;
(2) The linear and angular velocities in velocity space are sampled, allowing for the determination of the trajectory in time Δ*t* according to the magnitude and direction of the linear and angular velocities. The angle of the passable area is limited according to the trajectory, and the area with more obstacles is avoided as much as possible;
(3) The obstacle map is processed to identify the end position “T” and the start position “S”. Subsequently, global path planning is executed utilizing the enhanced hybrid genetic and ant colony algorithm for global path planning as described above;
(4) The globally optimal path is divided, and inflection points or obstacle occlusion points are selected for marking, designated as multi-level sub-target points;
(5) Concluding the dynamic planning of the environment construction map involves positioning the starting and ending points for the movement of dynamic obstacles. The dynamic obstacles are set to travel back and forth at a consistent speed (slower than the mobile robot’s movement speed), and new grey static obstacles are added;
(6) The passable area’s angle, which was determined in Step 2, acts as a constraint for the azimuth angle function. To expedite the algorithm’s convergence, the distance evaluation function takes the distance to the sub-target point into account. The velocity evaluation function takes into account the velocity differential between the mobile robot and the dynamic obstacle. This ensures a gradual reduction in velocity as the mobile robot approaches the dynamic obstacle, preventing a complete halt in movement. This strategy aims to smooth out the planned path and eliminate breakpoints;
(7) The three evaluation functions are normalized, and the linear and angular velocities in Δ*t* time are chosen based on the evaluation functions to complete the local obstacle avoidance and path planning. The planned path in a sub-interval is completed continuously for one segment until it reaches the sub-target point;
(8) After successfully avoiding dynamic obstacles during motion, the mobile robot promptly undergoes positional adjustments to return to the globally optimal path as soon as possible;
(9) Upon concluding the path planning for one sub-interval, Steps 6, 7, and 8 are continued to complete the local dynamic obstacle avoidance and path planning of the next sub-interval until the global path planning is completed and the target point location is set.

### 3.3. Global Optimal Paths Are Introduced

The local dynamic obstacle avoidance effect of the improved dynamic window method has been improved to some extent. However, inherent algorithmic limitations persist, making it susceptible to falling into the local optimum. Therefore, the global optimal path must be introduced to guarantee both the local dynamic obstacle avoidance and the superiority of the global planning path.

#### 3.3.1. Key Sub-Goal Point Setting

The dynamic window method in path planning lacks the overall consideration of global information, which leads to the inability of the algorithm to choose the correct “escape” path once the mobile robot encounters semi-enclosed obstacles such as “L”- and “C”-type paths [44]. Therefore, the critical target point method is introduced to divide the global planning path into several small segments according to the position of the obstacles for local dynamic planning, which can achieve better local obstacle avoidance and solve the disadvantage of poor global planning effect on the dynamic window method. The global optimal path is obtained by the A* algorithm. The global optimal path is divided according to the information on obstacle distribution location and corners. Then, the more representative path points are selected as the key sub-target points in the process of local planning by the dynamic window method.

Selection principle of key sub-target points:

(1) Corner principle: Emphasizing more prominent or critical corners and utilizing them as sub-target points to prevent the dynamic window method from losing targets in the planning process, resulting in the loss of planning effects;
(2) Obstacle avoidance principle: In cases of a longer linear running path, considering the distribution of obstacles, it is prudent to opt for path points that are distant from obstacles as sub-target points. This strategy helps minimize the likelihood of collisions with obstacles.

#### 3.3.2. Movement Position Adjustment

**Figure 5:**
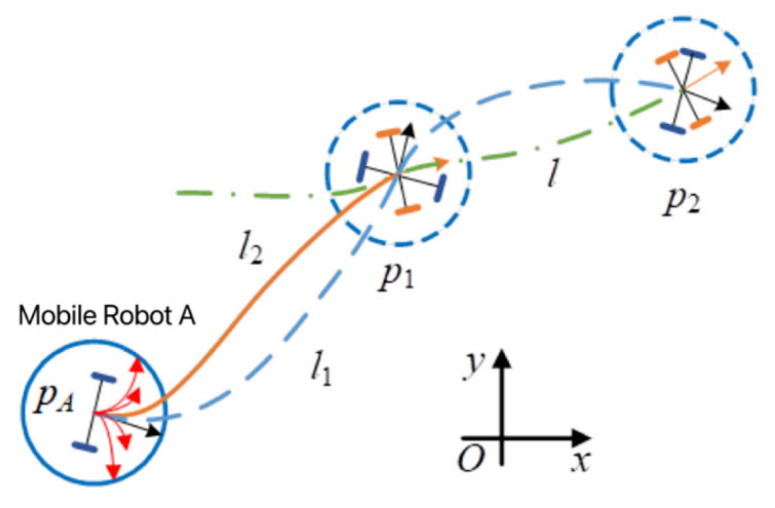
Mobile robot motion posture adjustment.

A discrepancy exists between the positioning orientation at the sub-goal point and the positional attitude of the globally planned path. This occurs due to the dynamic window technique unavoidably departing from the global ideal path during the dynamic planning process. It becomes increasingly challenging to follow the intended global optimal path as this gap widens. To ensure that the mobile robot’s trajectory realigns with the overall ideal path while avoiding dynamic obstacles, the position attitude of the robot must be changed in real time. Figure 5 illustrates how the mobile robot’s mobility stance can be adjusted.

The arrowed lines in Figure 5 represent the amount and direction of potential movement velocities. The brown solid line *l*_2_ is the planning path after setting multi-level sub-target points, from position *p*_1_ to position *p*_2_, close to the green dashed line *l*_1_, enabling position adjustment during robot movement. The blue dashed line *l*_1_ is the planning path of the improved dynamic windowing algorithm in this section. It is the total error brought on by the mobile robot’s departure from the intended path once dynamic obstacle avoidance has been successful.

## 4. Simulation of Indoor Dynamic Environment Simulation Experiments

### 4.1. Experimental Parameter Setting of Dynamic Window Method

The improved dynamic windowing algorithm proposed in this section is compared with the A* fusion DWA algorithm proposed by Pang et al. [45], and simulation experiments are performed in different dynamic environments using MATLAB R2021b.

In the simulation environment, the computer system environment is Windows 10, the processor is Intel Core i7-12700H (Intel, Santa Clara, CA, USA), the running memory is 6 GB, and the compilation environment is MATLAB2018b. The relevant parameters of the dynamic window method are set as shown in Table 2.

The initial pointing weights (azimuth weights), initial safety distance weights, initial velocity weights, and maximum velocity weights are set to prevent the velocity weights from being too large and causing the mobile robot to miss the target point due to the large velocity share in the movement process.

### 4.2. Simulation Comparison Experiment under the Dynamic Environment of Unidirectional Motion

The decision has been made to compare and analyze the motion trajectory graph, attitude angle against time graph, linear velocity and angular velocity against time graph, dynamic window algorithm, A* fusion dynamic window algorithm, and improved dynamic window method. The small yellow square that is the dynamic obstacle moves in one direction at a constant speed of 1 m/s, as shown by the dotted line. The black square is a stationary barrier with known dimensions. The green straight line is the motion guide, and the blue one is the program trajectory.

**Figure 6:**
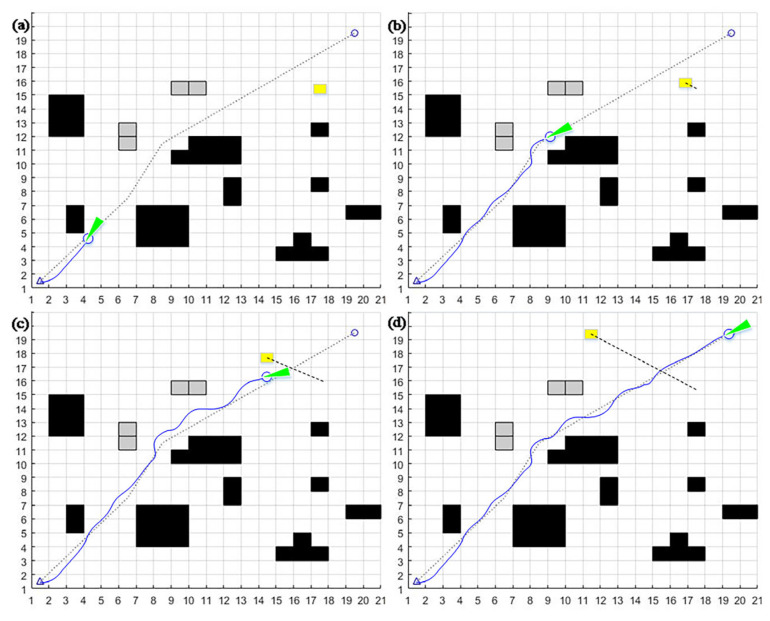
DWA algorithm one-way motion trajectory in a dynamic environment: (**a**) avoid known static obstacles; (**b**) avoid new static obstacles; (**c**) detect dynamic obstacles; (**d**) successfully avoid dynamic obstacles and reach the endpoint.

**Figure 7:**
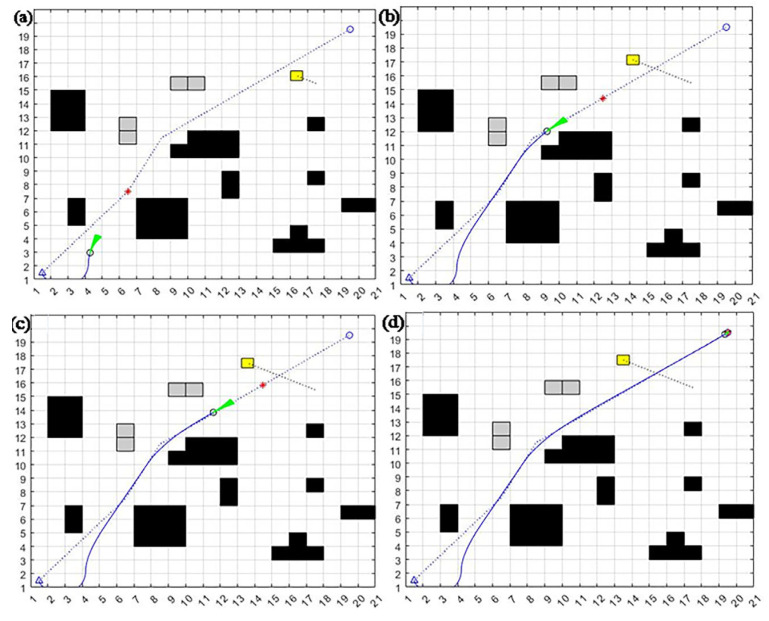
A* fusion DWA algorithm one-way motion dynamic environment obstacle avoidance motion trajectory: (**a**) follow the first sub-target point, avoid static obstacles; (**b**) follow the second sub-target point; (**c**) follow the third sub-target point, detect dynamic obstacles; (**d**) successfully avoid dynamic obstacles, reach the endpoint.

**Figure 8:**
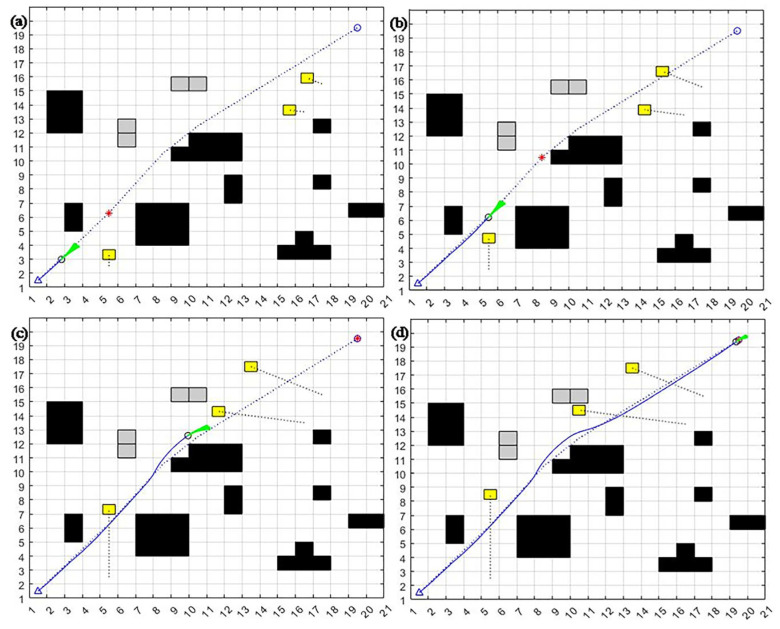
Improvement of the trajectory of the DWA algorithm for obstacle avoidance in a one-way motion dynamic environment: (**a**) detect the first dynamic obstacle; (**b**) accelerate to avoid the first dynamic obstacle; (**c**) detect the second dynamic obstacle; (**d**) successfully avoid all dynamic obstacles and reach the endpoint.

From the comparison of Figure 6, Figure 7 and Figure 8, it can be seen that although the dynamic window method can achieve dynamic obstacle avoidance, the planning path is too tortuous. The A* fusion DWA algorithm can achieve dynamic obstacle avoidance in simple static and one-way motion dynamic environments. However, due to the problem of the way the A* algorithm searches for path points, it can cause the algorithm to deviate too much from the original global path at the beginning, increasing path length. The improved dynamic window algorithm proposed in this section can achieve good obstacle avoidance for new static and multi-dynamic obstacles with unidirectional motion. It can return to the global planning path soon after local obstacle avoidance. Thus, it maintains a better overall path and saves time.

**Figure 9:**
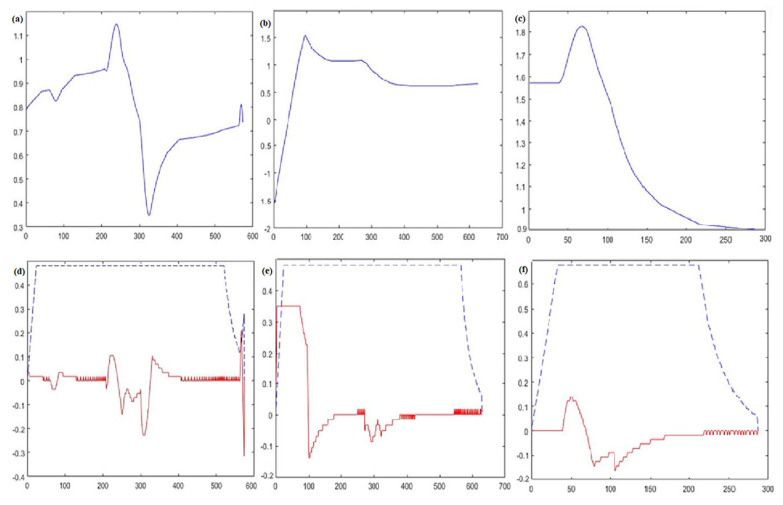
The attitude angle versus time curves are (**a**–**c**), and the linear and angular velocity versus time curves are (**d**–**f**). (**a**,**d**) DWA algorithm; (**b**,**e**) A* fusion DWA algorithm; (**c**,**f**) improved DWA algorithm. The solid blue line represents the orientation angle, the dashed blue line represents the linear velocity, and the solid red line represents the angular velocity.

The comparisons in Figure 9 highlight that improved dynamic window algorithm proposed in this section has a smoother change in attitude angle compared to the A* fusion DWA algorithm. It allows the mobile robot to reduce the rotation angle during motion, ultimately reducing energy consumption.

Regarding line velocity and angular velocity variation, the improved dynamic window algorithm has a lower line velocity and angular velocity variation. This characteristic demands less frequent and intense acceleration and deceleration, thus ensuring the operation of the mobile robot. The three algorithms’ average planning time and optimal path length are statistically recorded and compared for analysis.

The average planning times of the DWA algorithm, A* fusion DWA algorithm, and improved DWA algorithm in a dynamic environment are 64.58, 56.70, and 48.84, respectively, and the average path lengths are 34.57, 32.22, and 31.02, respectively.

From the above results, it is clear that the improved DWA algorithm proposed in this section reduces the average planning time by 25.6% and 13.8% and the average path length by 10.2% and 3.7%, respectively, compared to the dynamic window method and the A* fusion DWA algorithm in a one-way motion dynamic environment.

### 4.3. Simulation and Comparison Experiments under the Dynamic Environment of Round-Trip Motion

To further validate the effectiveness of the algorithm, a simulation comparison experiment is conducted again under the dynamic environment where the obstacles perform round-trip motion. The new static obstacle location and number and dynamic obstacle starting point, endpoint, and trajectory are different, and dynamic obstacles’ uniform round-trip motion, algorithm settings, and other simulation experimental conditions are kept the same.

**Figure 10:**
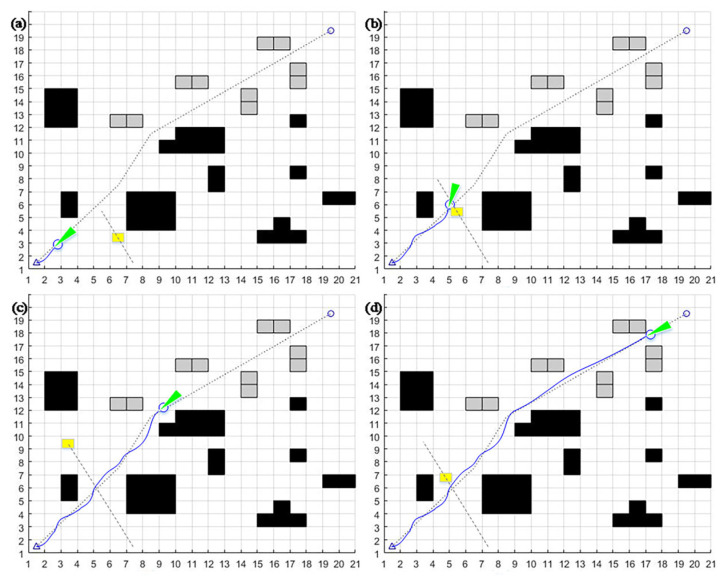
DWA algorithm round-trip motion trajectory in a dynamic environment: (**a**) detect dynamic obstacles; (**b**) hit dynamic obstacles; (**c**) avoid new static obstacles; (**d**) avoid all static obstacles.

**Figure 11:**
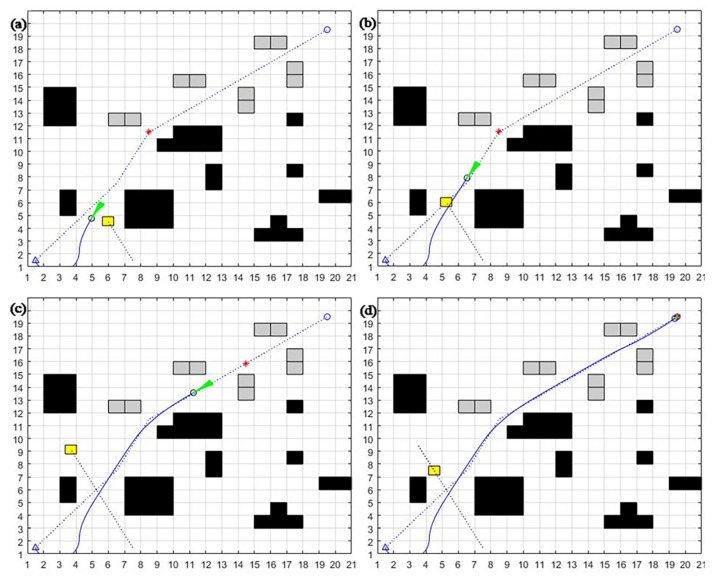
A* fusion DWA algorithm round-trip motion dynamic environment obstacle avoidance motion trajectory: (**a**) detect the first dynamic obstacles; (**b**) accelerate to avoid the first dynamic obstacles; (**c**) follow the second sub-target point to avoid additional static obstacles; (**d**) successfully avoid all static and dynamic obstacles and reach the endpoint.

**Figure 12:**
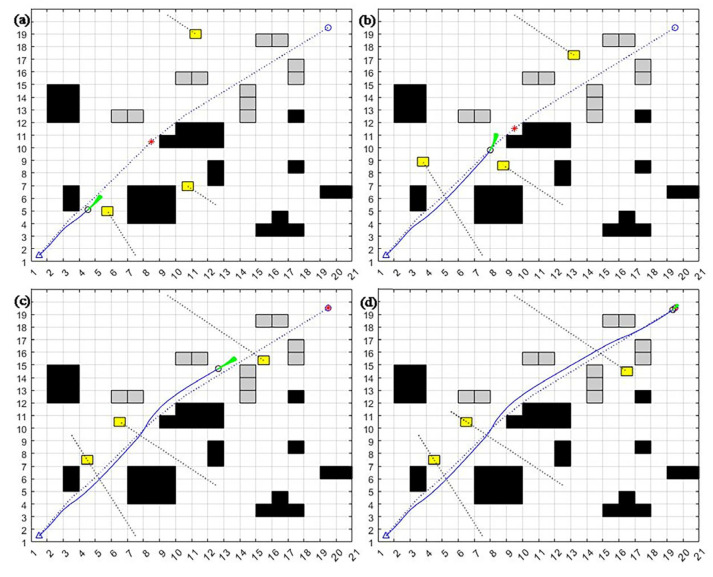
Improvement in the DWA algorithm round-trip motion trajectory in a dynamic environment: (**a**) detect the first dynamic obstacle; (**b**) detect the second dynamic obstacle and accelerate to avoid it; (**c**) detect the third dynamic obstacle; (**d**) successfully avoid all static and dynamic obstacles and reach the endpoint.

From Figure 10, Figure 11 and Figure 12, it can be seen that the dynamic window method cannot achieve good dynamic obstacle avoidance when the environment becomes more complex. It will be easy to fall into the local optimum or even hit dynamic obstacles.

The A* fusion DWA algorithm can achieve static obstacle avoidance. However, when facing dynamic obstacles, the algorithm will deviate too much from the original global path at the beginning, leading to an increase in path length and wastage of time. The improved dynamic window algorithm presented in this section can better achieve the obstacle avoidance of new static and multiple dynamic obstacles. It can slowly return to the global optimal planning path by adjusting the position after the local obstacle avoidance to keep to the overall path better and save time.

**Figure 13:**
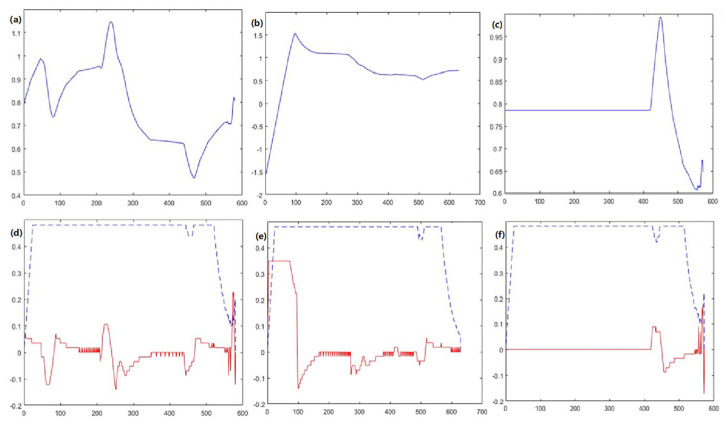
The attitude angle versus time curves are (**a**–**c**), and the linear and angular velocity versus time curves are (**d**–**f**). (**a**,**d**) DWA algorithm; (**b**,**e**) A* fusion DWA algorithm; (**c**,**f**) improved DWA algorithm. The solid blue line represents the orientation angle, the dashed blue line represents the linear velocity, and the solid red line represents the angular velocity.

As can be seen in Figure 13, compared with the dynamic window algorithm and the A* fusion DWA algorithm, the improved dynamic window algorithm proposed in this section has a smoother change in attitude angle. It makes the mobile robot not rotate as much during the motion, thus making the operation process smoother. Concerning line velocity and angular velocity variation, the improved dynamic window algorithm has lower line velocity and angular velocity variation, which can ensure the smooth operation of the mobile robot.

The three algorithms’ average planning time and optimal path length are statistically recorded and compared for analysis. The average planning times of the DWA algorithm, A* fusion DWA algorithm, and improved DWA algorithm in a dynamic environment are 66.23, 51.88, and 49.57, respectively, and the average path lengths are 38.73, 37.45, and 36.63, respectively.

From the above results, it is clear that the improved DWA algorithm reduces the average planning time by 22.6% and 4.5% and the average path length by 3.3% and 2.2%, respectively, compared to the dynamic window method and the A* fusion DWA algorithm in the complex dynamic environment.

From the above simulation comparison experiments as well as data comparison, it can be concluded that the improved dynamic window algorithm in this section has better results in terms of path length, planning time, motion trajectory, and smooth motion compared with the dynamic window method and A* fusion dynamic window method. It can make the mobile robot’s obstacle avoidance safer and more effective, the planning path smoother, and the mobile robot’s running state smoother. Consequently, it holds significant advantages in local path planning and obstacle avoidance.

## 5. Conclusions and Future Work

We concentrate on the analysis of obstacle avoidance in this research within the setting of the actual dynamic motion of mobile robots. Fuzzy control is integrated with the dynamic window technique. To optimize the dynamic window method’s assessment sub-function and enhance its potential for safe and reliable dynamic obstacle avoidance, the combination of weights is changed using fuzzy logic. Next, a vital point is chosen on the global path. These selected crucial points are employed as reference sub-target points to keep out of local optima while the improved dynamic window approach is in motion. After avoiding local obstacles, the mobile robot’s position is swiftly changed to stay on the global ideal path. Through experimental validation, the improved dynamic window algorithm suggested in this paper outperforms the dynamic window algorithm and the A* fusion DWA algorithm in terms of the mobile robot’s ability to avoid obstacles and plan globally in a variety of dynamic environments.

## Figures and Tables

**Table 1:** Fuzzy rule table.

Rule Serial Number	Logic Inputs		Logic Outputs		
	Dist-Goal	Dist-Obstacle	*α*	*β*	*γ*
1	PS	PS	PB	PM	PS
2	PS	PM	PB	PM	PS
3	PS	PB	PB	PS	PS
4	PM	PS	PS	PB	PS
5	PM	PM	PM	PM	PS
6	PM	PB	PM	PS	PB
7	PB	PS	PS	PB	PS
8	PB	PM	PM	PM	PM
9	PB	PB	PB	PS	PB

**Table 2:** Parameters related to the dynamic windowing algorithm.

Parameters	Numerical Value
Obstacle conflict radius determination R_1_/m	0.5
Local target point radius determination R_2_/m	1.5
Time resolution/(dt/s)	0.1
Linear speed resolution/(m/s^−1^)	0.01
Angular velocity resolution/(°/s^−1^)	1
Forward prediction time/(t/s)	3
Initial pointing weights	0.08
Initial safety distance weights	0.2
Initial velocity weights	0.1
Maximum speed weights	0.3

## Data Availability

The clerk and the code mentioned in the article are still needed for subsequent use and cannot be made available at this time.
